# Malarial Hæmaturia

**Published:** 1892-03

**Authors:** E. H. M. Parham

**Affiliations:** Fordyce, Ark.


### MALARIAL HÆMATURIA.

BY E. H. M. PARHAM, M. D., FORDYCE, ARK.

ANSWER TO DR MCHATTON.

Without wishing to cause Dr. McHatton to continue the discussion of the above important subject longer, or attempting to convince him of an error, to which, perhaps, an over zeal for science may have led him, I have concluded to notice briefly a few remarks contained in his last article. He stated that I said that I controlled the hæmorrhage and altered the abnormal condition of the blood with ergotole, tinct. iron, aa 10 gtts., which, as far as he could see, is an absolute assertion with no opening for escape. I beg leave to assure the doctor that I have no desire to escape from that assertion, but rather repeat it; he also says that the evacuant pleasantry was the result of a typographical error in my first article. In that he is mistaken; the typographical error might have been in his, certainly not in mine, as I had nothing to say about it.

It is strange indeed, that the doctor should discredit my diagnosis, on account of the gratifying results of my treatment, when I had already accepted his symptomatology as correct; there certainly can be no mistake about that. Simple hæmaturia is a disease entirely different from the one under discussion, and it is those with continued fever and other concomitant symptoms alone that we call malarial hæmaturia. I named competent references in a previous article, who are perfectly conversant with the subject under discussion, and probably one or more of them will express their views through the SOUTHERN MEDICAL RECORD before a distant day. The doctor also says that mistaking hæmoglobinuria for hæmaturia was the cause that the disease was erroneously treated; certainly, the discovery cannot be attended with much advantage, since he acknowledges that the mortality has never been less than thirty per cent. While I delight in scientific investigation as well as the doctor, I care very little whether it is the one or the other, provided my efforts are attended with as good results as they have been heretofore.

I have long since believed that it was the duty of every practicing physician to express his views freely, in a fraternal spirit, in regard to articles which he may think misleading. We are dealing directly with human life and can never recover that which is lost. It was that conviction that prompted me to write what I have; having redeemed what I conceive to be a sacred obligation, and one too that I can never regret, I beg leave to withdraw from further comment, so far as I am individually concerned.

What I have had to say was the result of my own experience, without quoting the experience of others. I might have compiled the experience of others with but little trouble, but any first-course student could have done that. The doctor will excuse me for the last sentence, as I have studiously avoided slang of every kind.

It might not be out of place in this connection to give a brief account of what occurred in relation to the use of peruvian bark, also the use of quinine as soon as separated from the other principles of the bark and having gone into general use, from the year 1830 to the year 1840. I regret very much that I have to refer in part to information obtained through the kindness of two of my elder brothers, (Drs. Thomas & Wm. L. Parham) both of whom were graduates of the University of Pennsylvania. I did an extensive practice in Greensville and Nansemond counties, Va., respectively. Brother William lived and practiced medicine in Nansemond county three or four years and died. I commenced studying medicine with him; Brother Thomas practiced that entire decade in Greensville county, Va., and I do not know two counties that furnish more evidence of malaria than those, and yet the disease called malarial hæmaturia or malarial hæmoglobinuria, was not known in those counties, during that entire decade; and at the same time, peruvian bark and quinine as soon as it could be had was used with the utmost caution, never prescribing either until the patient was thoroughly prepared with a complete intermission of fever.

This state of thing continued some time after that; but after a while quinine became generally used in all states and conditions of malarial troubles by the people generally, without preparing the patient for its use, and without a knowledge of any evil consequences which might arise from its abuse; and the consequence was, that malarial hæmaturia or malarial hæmoglobinuria appeared in a violent form and was very fatal to those treated with quinine, and unfortunately, from what I have been able to learn, a large majority were treated in that way.

I have made a simple statement of what I know to be facts and leave it to the members of the medical profession to draw such conclusions as their better judgment may direct.

I have my own convictions about it and will act accordingly; others of course will do as they like.

I repeat that I regret very much the necessity of refering to my brothers, and having faithfully performed what I conceive to be my duty, I willingly withdraw from further discussion of the above subject with Dr. McHatton, with no unpleasant recollections of him, believing as I do that it will be attended with good results, inasmuch as it is calculated to cause members of our profession to be more watchful of the effects of remedies, especially quinine, which were the chief objects of my articles.

