# Errata

**Published:** 1892-04

**Authors:** 


ERRATA.—In the March issue we wish to call attention to the following error in the article of Dr. E. H. M. Parham on Malarial Hæmaturia: "Drs. Thos. and Wm. L. Parham, both of whom were graduates of the University of Pennsylvania. I did an extensive practice," etc., which should have read, "both of whom were graduates of the University of Pennsylvania, and did an extensive practice."

